# Illinois State Medical Society

**Published:** 1871-07

**Authors:** 


### Proceedings of Societies.

### ILLINOIS STATE MEDICAL SOCIETY.

The Annual Meeting of this Society was held in Peoria, commencing on Tuesday morning, May 16th, and continuing until one o'clock P.M. of the Thursday following. It was one of the largest and best meetings that have been held since the organization of the Society in 1850. The following record embraces the most important part of the proceedings:

The Society was called to order at ten o'clock, by the President, Dr. G. W. Albin.

The roll of officers was called, and all found to be present, as follows:

*President*—G. W. Albin, M.D., of Neoga.

*First Vice-President*—John Murphy, M.D., of Peoria.

*Second Vice-President*—J. S. Whitmire, M.D., of Metamora.

*Treasurer*—J. H. Hollister, M.D., of Chicago.

*Permanent-Secretary*—T. D. Fitch, M.D., of Chicago.

*Assistant-Secretary*—J. P. Johnson, M.D., of Peoria.

*Committee of Arrangements*—Drs. J. C. Frye, R. Boal, J. O. Hamilton, R. Roskoten, Peoria; S. Maus, Pekin; J. S. Whitmire, Metamora.

Drs. Peck and Baxter, delegates from the Iowa State Medical Society, were introduced by Dr. Plummer, of Rock Island, and were invited to take seats and participate in the business of the meeting as delegates. Dr. Peck responded briefly, and invited the Society to appoint delegates to attend the next annual meeting of the Iowa Society.

On motion of Dr. J. O. Hamilton, the annual assessment for each member was fixed at $3.00.

The hearing of reports from standing committees being in order, Dr. H. A. Johnson, of Chicago, chairman of the Committee on Practical Medicine, gave a verbal abstract of a somewhat lengthy and interesting report, the materials for which had been obtained chiefly from the records of the Chicago Board of Health.

At the close of the report a recess was taken for dinner.

AFTERNOON SESSION.

The Society was called to order at two o'clock P.M., President Albin in the chair.

Dr. Boal, in behalf of the Committee of Arrangements, delivered a brief and very appropriate address of welcome; and several additional members presented their credentials and were registered.

The consideration of the report of the Committee on Practical Medicine was resumed, and Dr. G. B. Hawley, of Aurora, inquired if the report contained anything in regard to the condition of certain classes of people in different parts of the city, the actual hygrometric condition of the atmosphere, as well as the amount of rain fall, etc.

Dr. H. A. Johnson stated that the report did contain details in regard to all the matters mentioned by Dr. Hawley, except that of the hygrometric condition of the atmosphere, and he stated some striking facts illustrating the effects of sewerage and pure water in lessening the mortality. He also stated that the south and south-east winds were always followed by an increase in the daily number of deaths in the city.

Dr. Hewett stated that in his locality in Jersey County he had observed an aggravation of disease pretty uniformly in connection with dry south and south-east winds. The subjects embraced in the report were further discussed by Drs. D. W. Young, of Aurora, J. H. Hollister, and N. S. Davis, of Chicago. The latter stated that temperature and moisture certainly exert a great influence in the production of diseases, but in the production of bowel-affections, so prevalent, especially among children in the summer, another element acted in conjunction with the heat and moisture. That element was some kind of atmospheric impurity. He had observed that the bowel-complaints of children in Chicago invariably commence with the first week of continuous hot weather after the fresh fallen surface water of spring has disappeared, so that the surface soil is simply moist, or in the process of becoming dry. It is then that the processes of organic change and decomposition are most rapid, and the products impregnate the atmosphere to the greatest extent.

Dr. J. C. Corbus, of Mendota, member of the Committee on Practical Medicine, made a supplementary report in relation to the diseases of his locality, which, together with the report of Dr. Johnson, was referred to the Committee of Publication.

The President then announced that the Mayor and City Attorney of Peoria were present, and desired an audience with the Society.

The Mayor introduced Mr. Quinn, the City Attorney, who said:

Mr. President and Gentlemen of the Convention: The City Council of our city, at a recent date, met and unanimously adopted a resolution in reference to your assembling here, whereby in behalf of our citizens they extended to you a hearty welcome and the hospitalities of the city, and directed that their action should be made known to you by the mayor. On account of his modesty, and at his request, the pleasing duty devolves upon me to extend to you the earnest, hearty welcome of our people. Therefore, in behalf of our city authorities and the 25,000 good people whom they represent, I extend to you, gentlemen of the medical profession—of that art above all arts, the healing art—a most cordial greeting, and a warm, unselfish, hearty welcome. And, gentlemen, this is not a mere formality; it is a genuine expression of an unselfish sentiment which finds its well-spring in the heart. We have no base motive, no unkind thought in extending to you our hands, welcoming you to our houses, and warming you in our hearts.

If you controlled legislation, represented the executive, the cabinet, the army or the navy, and we paid you deference and honor, it might be said that we were sycophants seeking favors from those having power to grant them, but you being neither one nor the other, this lies not upon the tongue of slander to be uttered.

But why, I may be asked, this stir, this earnest effort on the part of our people to make you at home—to make you forget that you are away from home? Simply this: We know that you are the benefactors of the human race; that you ameliorate suffering; give ease to the throbbing temple; quiet the excited pulse; and bring rest and sweet repose to the troubled pillow. We know that braver soldiers never stood on guard than you and your brethren, for no matter what the disease, the epidemic, the scourge, you never turn and flee, but bravely and boldly assail it.

In the velvet-clad palaces of the rich and the neglected cabins of the poor, there, gentlemen, we find you.

Exposed to storms and tempests, snows and sunshine, light and darkness, all to remove suffering, to heal the troubled breast. Hence, being mindful of the blessings you bring to us, and the sufferings you endure for us, we admire you, nay, love you, and are resolved to do all in our power to make your stay in our midst one to be remembered with pleasure, until the lamp of your existence is extinguished.

Gentlemen, before returning to your respective homes, we desire to have you look at our little city, and see if the Lord, at least, has not been good to us. And, as President Johnson once said, so I say, "Look at Peoria."

Our city, gentlemen, whether or not she receives what many of the people of our State hope and pray she may receive, will always have a beautiful lake, a half-a-dozen railroads, delightful scenery, brave men, and handsome women, good health, and as good physicians as any community has cause to be proud of.

Gentlemen, without reflection or forethought I offer these words, feeling how inadequate they are for the occasion, I having simply stolen a moment from professional labors to perform what to me is a pleasure and honor.

In conclusion, gentlemen, you are welcome, thrice welcome to Peoria.

Dr. Johnson, of Chicago, was called upon to reply to the speech in behalf of the Society. He had met with the Society for 21 years, and nowhere had the Society been treated so kindly as in this city. He was astonished to be so well treated. After going to a hotel and engaging a room, he found that he had been assigned a pleasant home while in this city, and had nothing to pay. He replied in brief to the remarks of Mr. Quinn, complementary to the profession, and its aims and objects. This Society would always remember with pleasure their visit to the city of Peoria, and he wished the city the greatest improvement and happiness.

Dr. Edmund Andrews, of Chicago, chairman of the Committee on Surgery, presented an interesting report on various surgical topics, embracing improvements in the operation for phymosis by Prof. J. S. Sherman, of Chicago; improved instruments for operating for cleft palate, irrigating the urethra, suppressing hemorrhage, using the endescope, the choice of anæsthetics, the effects of transplantation of skin and cuticle in the healing of chronic ulcers, etc. He related three cases of the latter process by himself, and alluded to one by Prof. E. Powell.

Dr. D. Prince, of Jacksonville, stated that he had made several experiments in the transplantation of cuticle to the surface of chronic ulcers. In all of his cases the ulcers were suppurative, and the experiments failed. He had been informed by Dr. Hodgen, of St. Louis, that his trials in ulcers of a similar kind had also failed, but when the transplantation had been to the surface of ulcers that were dry or non-suppurative, it had been followed by a satisfactory degree of success.

Dr. Andrews, in his report, had recommended a row of gas jets ten inches long for illuminating the urethra and other internal parts, and Dr. H. A. Johnson inquired whether the color of the light needed any correction in order to appreciate correctly the color of the illuminated surfaces?

Dr. Andrews replied that the light was sufficiently accurate for practical purposes.

Dr. Johnson added that he had found an ordinary Argand burner with a deep blue chimney to give the color of tissue very perfect. He also stated that recent cases of gonorrhœa could be generally rendered abortive, or speedily cured, by the local application of a solution of permanganate of potassa, 10

grs. to the oz. of water. The application is unirritating and painless.

Dr. D. Prince said that tannic acid, moistened with glycerine and made into the form of a bougie, when introduced into the urethra and left to dissolve in the moisture of the mucous membrane, generally rendered recent gonorrhœa abortive.

Dr. Kilbourn spoke against the use of ordinary astringents in the treatment of this disease, and recommended the method of irrigation described in the report of the committee.

Dr. T. D. Fitch thought the method of operating for phymosis adopted by Dr. J. S. Sherman, as described in the report, was not very different from that recommended by Erichsen, in his work on surgery, and which he had followed in his own practice with entirely satisfactory results. In regard to the use of anæsthetics in surgery, he stated that up to the last two years he had given the preference to chloroform, and had so expressed himself in the anniversary meeting of this Society for 1869. But since that time his personal observations, as well as the accumulated facts in our medical literature, had induced a change in his mind, and he now thought chloroform should be used only in exceptional cases. He still entertained fears that though ether was much less dangerous in its immediate effects, yet its secondary influence on the blood might materially retard or aid in preventing the final recovery of the patient.

Dr. Harriot inquired how Drs. Fitch and Andrews administered chloroform, and was informed that they used it on a napkin over the mouth and nostrils of the patient, holding it in such position as to allow a reasonable admixture of atmospheric air. He stated that he had used chloroform very often for a number of years without any bad results.

Dr. J. H. Hollister inquired whether any one had known fatal results from the inhalation of chloroform after the preceding inhalation of ether?

Dr. Mosher said that Dr. Frank H. Hamilton, of New York, gave ether to a patient, and failing to produce the desired anæsthesia, followed it by chloroform, which proved speedily fatal.

Dr. Traverse had given chloroform frequently in obstetric practice without any dangerous effects, but thought there was more danger from hemorrhage after the delivery; so much so, that he now pretty generally gave a dose of ergot immediately after the completion of the labor.

Dr. White, of Bloomington, also thought the use of chloroform increased the tendency to excessive flowing after labor. He related a death from the inhalation of chloroform used by mistake, supposing it to be ether. The *post mortem* showed the blood to be in a fluid condition, and a specimen of it kept in a vial several months presented no coagulation.

Dr. H. A. Johnson alluded to a fatal case of poisoning by hydrate of chloral, recently published in Europe, in which the *post mortem* revealed the presence of chloroform in the blood, and made some comments on the philosophy of anæsthesia.

Dr. Crawford said he had used chloroform as the principal anæsthetic in his practice, using it often, and without any serious results. He thought much of the safety or danger incurred in its use depended on the mode of its administration. The method which he learned while connected with the army was to lay one or two thicknesses of factory cloth over the mouth and nostrils of the patient and then put the chloroform on it drop by drop.

Dr. J. S. Whitmire practised and recommended the same method, and though he had given the chloroform a great many times, and still adhered to its use, he had met with no bad results. He believed, however, that its use should be limited to really important cases of surgery, and strongly condemned its use for extracting teeth and other trivial operations.

Dr. S. J. Jones and Dr. W. Young coincided with Drs. Crawford and Whitmire in relation to the method of using chloroform. The latter gentleman stated that the reason why so few accidents had resulted from the use of chloroform in obstetrics compared with general surgery was, because in labor it is given to allay pain already present, while in surgery it is given in advance, to prevent suffering anticipated.

Dr. Jenks stated that he had found a mixture of chloroform, tinct. opii., and tinct. aconite, applied locally to the gums, to produce all the anæsthesia necessary to extracting teeth.

On motion, the report of Dr. Andrews on surgery was referred to the Committee of Publication.

The Society then adjourned until 8 o'clock P.M.

EVENING SESSION.

On re-assembling at 8 o'clock in the evening, Dr. Albin in the chair, Dr. J. Murphy, of Peoria, made an interesting verbal report concerning his method of treating strictures of the urethra. For all old and indurated strictures he recommended the use of chromic acid, applied directly to the stricture. He introduces a large-sized bougie, with several holes in the end, presses it down upon the stricture and injects through it the solution of chromic acid, repeating the application at first twice a day. When the tissue of the stricture has been softened by the application, a small-sized bougie, with holes in the sides, is used and pressed through the stricture, and the acid injected as before. He continues this once a day, with increased size of the instrument, until a No. 8 passes readily. He commences the use of a solution of chromic acid, 40 grs., water, ℥j., but increases the strength gradually to 60 grs. to the ounce of water.

Dr. D. Prince, of Jacksonville, member of the Committee on Surgery, made an interesting and lengthy report on the improvements in the departments of plastic and orthopœdic surgery, which was referred to the Committee on Publication.

The Committee on Obstetrics and Diseases of Women, Dr. DeLaskie Miller, of Chicago, chairman; and the Committee on Criminal Abortion, Dr. W. H. Byford, of Chicago, chairman, were called, but no reports were made.

The Committee on Ophthalmology, Dr. H. H. Roman, of Springfield, chairman, was called, but no report from the chairman. Dr. E. L. Holmes, of Chicago, one of the committee, read a short report on cataract, which was referred to the Committee of Publication.

The Society then adjourned until eight o'clock the next morning.

WEDNESDAY MORNING.—SECOND DAY.

The Society was called to order at 8 o'clock A.M., Dr. G. W. Albin, in the chair.

Dr. Boal, chairman of the Committee on Arrangements, reported that the committee had provided a new meeting place in the basement of the Universalist Church.

Dr. Davis moved that the meeting adjourn to the new rooms selected, but withdrew his motion, as it was desirable to remain in the hall to witness the exhibition of Dr. Truesdale's fracture-bed, after which the Society adjourned to the lecture-room of the Universalist Church. The new place of meeting is much more suitable than Rouse's Hall was. The accoustic qualities of the room are better; it is not so large, and therefore better suited to the use of the 100 or 150 delegates, and the seats and general appointments are much more comfortable. The Society were compelled to change their place of meeting on account of a minstrel performance.

The following names were proposed for permanent membership: Drs. J. P. Walker and N. Elsbury, of Mason City, Ill., by Drs. D. Prince and E. Andrews; Dr. C. D. Knapp, of San Jose, and Dr. N. B. Cole, of Bloomington, by Drs. R. D. Bradley and J. S. White; and Dr. John Gregory, by Drs. J. W. Hensley and H. Steele.

On suggestion of Dr. Davis, a recess of ten minutes was taken to receive the names of the nominating committee. When the meeting was called to order, the Committee on Nominations reported.

Dr. David Prince, of Jacksonville, then introduced to the Society Drs. Wm. M. McPhelters and Wm. S. Edgar, of St. Louis, and moved that they be allowed the rights and privileges of delegates, which motion was unanimously adopted.

Dr. Johnson, of Peoria, continued the report on opthamology, in the case of ocular hyperæthusia, which was discussed by Drs. Warner, McFarland, Johnson, Littlefield, and Holmes, and on motion, the matter was referred to the Committee on Publication.

The Committee on Phthisis Pulmonalis was called, when it was announced by the President that the Chairman, Dr. Noble, was dead.

The Committee on Otology was called, and the Chairman, Dr. S. J. Jones, of Chicago, made his report, which was discussed by Dr. E. L. Holmes.

On motion, the report of the Committee on Otology was accepted, and referred to the Committee on Publication.

The Committee on Investigation reported favorably on a number of names, presented as permanent members, and they were declared elected.

The Committee on Nominations made the following partial report:

Dr. J. O. Hamilton, of Jerseyville, President; Dr. T. Worrel, of Bloomington, First Vice-president; Dr. D. W. Young, of Aurora, Second Vice-president; Dr. J. H. Hollister, of Chicago, Treasurer.

On motion, this report was adopted, and the candidates elected unanimously, and on further report of the committee, Rock Island was selected as the next place of meeting.

The Committee on Criminal Insanity being called, Dr. McFarland, the Chairman, submitted a verbal report, which was discussed by Drs. McCartney, of Young America, and N. S. Davis, of Chicago.

The Committee on Idiocy was called, and Dr. C. T. Wilbur, of Jacksonville, presented his report, which on motion was adopted, and referred to the Committee on Publication.

On motion, the Society then adjourned.

In the afternoon, an excursion by rail was taken to Prospect Hill. Owing to the late railway war, and the blocking up of track, the train did not get away until nearly four o'clock, though the time announced for its departure was two o'clock. The Committee of Arrangements did all they could to hurry the departure, but could not control the matter, and are not to blame for the delay. Though the medical gentlemen were compelled to wait for a time at the foot of Hamilton Street, they took the matter good naturedly.

The number of citizens accompanying the excursion was limited. The train stopped at the residence of R. M. Cole, Esq., where three fine photographs were taken of the Society. This also consumed time, and the stay at Prospect Hill was necessarily very short. It gave the visitors, however, an opportunity of obtaining a fine view of the country from the hill, and many were the exclamations of pleasure at the prospect presented.

In the evening, a reception and banquet was held at Parmely's Hall. A large number of ladies and gentlemen were present, together with all our medical visitors. The banquet was prepared by the ladies of the city, and was worthy the attention it received. Spencer's band was in attendance, and dancing formed a pleasing feature of the evening. The company did not retire until a late hour, and all appeared to be well pleased with the entertainment.

THURSDAY MORNING.—THIRD DAY.

The Society was called to order at 9 A.M.; President Albin in the chair.

The reading of the minutes was dispensed with, and the regular order of reports of committees proceeded with.

Dr. N. S. Davis, chairman of a special committee read a report on the internal use of carbolic acid in the treatment of diseases, which was referred to the Committee of Publication.

Dr. D. W. Young, Special Committee on Cholera Infantum, read a lengthy and interesting report on that subject, which was referred to the Committee of Publication.

Dr. T. D. Fitch, Special Committee on Bowel Affections of Children, gave a verbal statement of his report on the nature and treatment of constipation in infants. The statement was accepted, and the author requested to furnish his report to the Committee of Publication. All these reports were listened to with much interest, and would have elicited very profitable discussion had not the hour for final adjournment been so near at hand.

Dr. J. S. Whitmire, Special Committee on the Duty of the Profession towards the State Medical Society, read a report, which was accepted, and referred to the Committee of Publication.

A paper on Criminal Abortion, by Dr. A. Niles, of Quincy, had been received through the Special Committee on that subject. The author not being present to read his paper, it was referred to the Committee of Publication, with instructions to examine the same and publish or not as they thought best.

The annual reports of the Treasurer and of the Publication Committee were presented, accepted, and placed on file.

They showed the financial condition of the Society highly satisfactory and prosperous.

Delegates were then elected to represent the Society in the next annual meeting of the American Medical Association, as follows:

Dr. N. S. Davis, Chicago.
Dr. F. B. Haller, Vandalia.
Dr. J. P. Johnson, Peoria.
Dr. M. W. Walton, Ridott.
Dr. J. O. Hamilton, Jerseyville.
Dr. D. M. Vosburgh, Earlville.
Dr. Geo. J. Monroe, Leland.
Dr. D. W. Young, Aurora.
Dr. E. P. Cook, Mendota.
Dr. J. L. White, Bloomington.
Dr. E. Willard, Wilmington.
Dr. C. Winney, Sandwich.
Dr. John Murphy, Peoria.
Dr. D. W. Jones, Danville.
Dr. David Prince, Jacksonville.
Dr. J. H. Hollister, Chicago.
Dr. C. Truesdale, Rock Island.
Dr. H. A. Johnson, Chicago.
Dr. E. Andrews, Chicago.
Dr. D. E. Foote, Belvidere.
Dr. T. D. Fitch, Chicago.
Dr. S. P. Hawley, Aurora.
Dr. N. G. Blalock, Mount Zion.

Dr. D. S. Jenks, Plano.
Dr. Lucius Clark, Rockford.
Dr. W. M. Kaul, Limerick.
Dr. E. M. Travers, Amboy.
Dr. Geo. W. Wright, Canton.
Dr. C. Goodbrake, Clinton.
Dr. Andrew McFarland, Jacksonville.

Delegates were also elected to the State Medical Societies of the adjoining States of Iowa, Missouri, and Indiana, as follows: To Missouri, Drs. G. W. Hall, of Carthage; F. B. Haller, of Vandalia; J. O. Hamilton, Jerseyville; and T. J. Pitman. To Iowa, Dr. Samuel C. Plummer, of Rock Island; and to Indiana, Dr. Wm. H. Byford, of Chicago.

A number of resolutions were adopted tendering thanks to the Committee of Arrangements, the proprietors of the hall and church in which the meetings were held, to the railroads, to the city authorities and citizens generally of Peoria for the liberal and excellent arrangements made and executed for the accommodation of the Society during the meeting about to close. The thanks of the Society were also tendered to the retiring President, for the able and faithful manner in which he had discharged his official duties.

The President, Dr. G. W. Albin, responded briefly, and the Society adjourned, to meet in Rock Island on the third Tuesday in May, 1872.

The foregoing is not the official record of the proceedings of the Society, but simply notes of such matters as we thought most interesting to our readers. If errors or mistakes have been made, the senior editor alone is responsible, and he will cheerfully correct any that may be pointed out.

The meeting was one of the largest and most profitable that has been held since the Society was organized. Yet it were easy to suggest improvements, which we may attempt to do in another place.

